# Distributed Spectrum Management in Cognitive Radio Networks by Consensus-Based Reinforcement Learning†

**DOI:** 10.3390/s21092970

**Published:** 2021-04-23

**Authors:** Dejan Dašić, Nemanja Ilić, Miljan Vučetić, Miroslav Perić, Marko Beko, Miloš S. Stanković

**Affiliations:** 1 Artificial Intelligence Department, Vlatacom Institute, 11070 Belgrade, Serbia; nilic@asss.edu.rs (N.I.); miljan.vucetic@vlatacom.com (M.V.); miroslav.peric@vlatacom.com (M.P.); milos.stankovic@singidunum.ac.rs (M.S.S.); 2 Faculty of Technical Sciences, Singidunum University, 11000 Belgrade, Serbia; 3 COPELABS, Universidade Lusófona de Humanidades e Tecnologias, 1749-024 Lisbon, Portugal; 4 Department of Information Technologies, College of Applied Technical Sciences, 37000 Kruševac, Serbia; 5 Instituto de Telecomunicações, Instituto Superior Técnico, Universidade de Lisboa, 1049-001 Lisbon, Portugal; beko.marko@ulusofona.pt; 6 Faculty of Information Technology and Engineering, University Union Nikola Tesla, 11158 Belgrade, Serbia

**Keywords:** multi-agent reinforcement learning, consensus algorithm, cognitive radio networking, joint spectrum sensing and channel selection, distributed policy evaluation, distributed Q-learning, off-policy temporal difference

## Abstract

In this paper, we propose a new algorithm for distributed spectrum sensing and channel selection in cognitive radio networks based on consensus. The algorithm operates within a multi-agent reinforcement learning scheme. The proposed consensus strategy, implemented over a directed, typically sparse, time-varying low-bandwidth communication network, enforces collaboration between the agents in a completely decentralized and distributed way. The motivation for the proposed approach comes directly from typical cognitive radio networks’ practical scenarios, where such a decentralized setting and distributed operation is of essential importance. Specifically, the proposed setting provides all the agents, in unknown environmental and application conditions, with viable network-wide information. Hence, a set of participating agents becomes capable of successful calculation of the optimal joint spectrum sensing and channel selection strategy even if the individual agents are not. The proposed algorithm is, by its nature, scalable and robust to node and link failures. The paper presents a detailed discussion and analysis of the algorithm’s characteristics, including the effects of denoising, the possibility of organizing coordinated actions, and the convergence rate improvement induced by the consensus scheme. The results of extensive simulations demonstrate the high effectiveness of the proposed algorithm, and that its behavior is close to the centralized scheme even in the case of sparse neighbor-based inter-node communication.

## 1. Introduction

A cognitive radio network (CRN) is an intelligent system of re-configurable wireless transceivers which can autonomously modify their configuration and communication parameters in order to meet quality of service (QoS) requirements or adapt to a changing network environment [1,2,3]. These modifications are achieved by incorporating several features in a cognitive radio device: (a) a cognition module (CM) at the software level, which provides intelligent decision-making, (b) advanced dynamic spectrum access (DSA) capabilities at the radio level, providing the ability to establish communication over various channels, and (c) low-bandwidth communication among typically neighboring transceivers, which enables cooperation. Network environment is locally sensed by the nodes of the CRN [4,5]. Based on the sensing information and the information communicated from the neighboring nodes, DSA capabilities enable the nodes to dynamically adjust transmission parameters according to the CM decisions. These adaptations to a changing environment enable dynamical reuse of vacant portions of the licensed (or unlicensed) spectrum by the CRN main actors (agents), the so-called secondary users (SUs) or unlicensed spectrum users, without affecting the performance of the frequency owners, the so-called primary users (PUs) [1]. PUs operate without consideration of SUs. Cognitive radios (CRs), acting as SUs, are realized usually as software defined radios [6]. In this context, inadequate sensing as a result of inability of an individual SU to successfully cover wide spectrum of frequencies, or its preference towards specific parts of the spectrum, may cause wasting spectrum opportunity and interference with PUs. In addition, the fact that channel sensing and quality assessment is prone to errors [5] may cause additional difficulties. Cooperation between the participating SUs can be a way of managing these challenges. In this respect, advanced machine learning techniques, such as reinforcement learning (RL), including cooperative schemes, have received much attention and treatice (see, e.g., References [2,7], and references therein).

RL can be used to treat general problems of decision-making in uncertain environments. In this approach, the environment is represented by a model of Markov decision processes (MDPs) [8]. RL methodology introduces the idea of a reward gained by the agent for actions taken, based on the influence that those actions cause to the environment observed. The goal of an agent is to maximize the long-term reward yield following a strategy or policy which defines actions to be taken in a certain state. Definition of policies employed in RL techniques are typically based on calculation or approximation of the state-value function or action-value function [8]. Off-policy learning is a setting of significant importance, especially in a distributed multi-agent scenario, in which participating agents evaluate a policy which differs from the policy applied for learning [9,10,11,12]. In addition, the multi-armed bandits (MAB) approach to decision-making, which can be treated as a separate field, but also as an RL framework focusing on exploration/exploitation trade-off in one-state MDPs, has found wide applications and gained a lot of popularity recently [13,14,15].

Multi-agent reinforcement learning (MARL) has been a popular research area for more than a decade [16]. An approach to decentralized and distributed MARL adopted in this paper is to allow agents to perform sparse communications through an inter-agent network (References [12,17] and references therein), assuming that the agents do not interact through the underlying MDP. The major appeal of this approach, which has gained popularity recently [18], can be found in the absence of any type of central coordinator (fusion center) and the fact that convergence and other properties of such an approach have been rigorously theoretically proved [11,12,17,18,19,20].

### Contributions

In this paper, we propose a novel distributed and decentralized algorithm for joint spectrum sensing and channel selection (JSS) in CRNs based on MARL with distributed consensus iterations over directed, possibly time-varying, communication graphs. Our main focus is to show that consensus schemes can be used as a valuable tool within the MARL approaches to solve problems arising in CRNs. We have published preliminary considerations related to these topics in the conference paper of Reference [21]. In contrast to the conference paper, we now propose two novel, previously unpublished algorithms for JSS, and thoroughly analyze their properties both in theory and through extensive simulations. To the best of the authors’ knowledge, there is no other previously published work regarding consensus-based MARL schemes in CRNs. In this paper, we consider both policy evaluation and policy optimization tasks. For policy evaluation, we apply consensus in parallel with the temporal difference (TD) learning algorithm [12,20]. We assume an off-policy setting, where the scheme is evaluating a single target policy, while the nodes/agents in the network are all implementing different behavior policies, according to their individual characteristics. For policy optimization, we propose to apply the consensus scheme to action-value function learning, where the agents are locally implementing the Q-learning algorithm with ϵ-greedy exploration/exploitation strategy in which each agent has a different set of channels of preference when exploring. This way, a complementary exploration can be achieved, increasing the general rate of convergence. In the limit, the channels of preference can be chosen such that, individually, the nodes do not have a possibility to successfully evaluate a policy or learn the optimal one, while collectively, using the proposed distributed scheme, this becomes feasible. It is assumed that the nodes communicate their current local estimates of the (either state or action) value function only with the neighboring nodes through low bandwidth communication links; hence, the setting is decentralized in the sense that each agent makes its own decisions, based only on local observations and information communicated with its neighbors, without the coordination of any central entity. Despite the decentralized nature of the algorithm, after a sufficient number of iterations, all the nodes in the network will have approximately the same estimate of the considered value function, as well as the optimal policy estimate in case of Q-learning applied. Another natural advantage of the proposed scheme, even when the selected behavior policies of the agents are not chosen to exploit complementarity, is the denoising effect which naturally arises when doing convexifications of the estimates in each consensus step of the learning process. Due to the nature of the consensus scheme, the algorithm is scalable and robust to nodes and links failures. All the convergence properties, introduced assumptions and limitations of the proposed algorithm have been analyzed and discussed. The extensive simulation results demonstrate the mentioned appealing properties and advantages of the introduced consensus-based scheme; even with a very sparse neighbor-based communication graph, the performance of the proposed scheme is very close to the globally optimal one. Numerical results also demonstrate the advantages of the proposed consensus-based scheme (relying on exchanging local value function estimates) over other representative cooperative RL schemes [2,22] (based on exchanging local rewards), due to the inherently higher variance of the value function estimates of the latter.

The rest of the paper is organized as follows. Section 2 presents an overview of the body of work related to the application of RL in the area of spectrum management in CRNs. Section 3 describes the problem setup and the CRN system model, as well as the corresponding Markov decision process framework. In Section 4, we introduce the proposed consensus-based TD off-policy state-value function approximation, as well as the consensus-based Q-learning scheme, and analyze and discuss their structural and convergence properties. Section 5 is devoted to the presentation of comprehensive simulation results, demonstrating applicability and high performance of the proposed scheme. Finally, Section 6 concludes the paper and gives future research directions.

## 2. Related Work

In the past decade, RL, especially MARL, has been established as a suitable paradigm for treating the spectrum management issues in CRNs. Different MARL algorithms that do not assume explicit cooperation between the agents have been proposed, focusing, e.g., on value function approximations [23,24], or MAB approaches [13,14], in the used learning schemes. Cooperative MARL schemes involving multiple fusion centers [1,25,26], or some form of centralization [27,28,29] have also been proposed. Some of these algorithms incorporate deep learning approaches, such as deep Q-networks (DQNs) [29]; the DQN approach has also been used in (single-agent) RL schemes [30]. Other RL schemes aim to predict how long the channel will remain unoccupied in addition to solving the channel selection task [31], or introduce novel hybrid spectrum access models [32]. Decentralized cooperative MARL algorithms based on comparing communicated information between the agents [22], or combining evolutionary game theory with DQNs [33] have also been proposed. To the authors’ best knowledge, the only approach to spectrum sensing in CRN based on dynamic consensus scheme was proposed in [34]; however, it treats a less general problem, not involving RL-based channel selection task. An overview of the RL-based approaches to CRN is given in Table 1.

## 3. Problem Description and System Model

The problem description and system model will be introduced through consideration of JSS task, introduction to RL and MDP and, finally, MDP formulation of the JSS problem.

### 3.1. Joint Spectrum Sensing and Channel Selection

The initial task, needed to be performed by SUs in order to identify the available spectrum resources for their use, is spectrum sensing. In practice, it is of great benefit to develop cooperative schemes which seek to aggregate channel sensing information from multiple SUs using minimal, neighbor-based communication requirements. The next task is dynamic channel selection (DCS). This task entails all of the SUs in a CRN setting performing spectrum selection while considering the interference caused to the primary spectrum owners, which must be minimized, and their own performance, which must be maximized. Both of the tasks described can be considered as part of the JSS task, which needs to be performed continuously and as quickly as possible at each node in order to adapt to the an ever changing environment. Exploiting cooperation is of essential importance for achieving this.

In what follows, we describe the adopted CRN system model, which is typically used in the existing literature with RL-based CRN (e.g., Reference [2]). We assume *N* SUs that operate within the same sensing domain, thus forming a CRN. DSA module of each CR (i.e., SU) enables environment observation and change of the operative frequency/channel, which is the transmission parameter of interest in the adopted setup. The licensed band which coincides with a set of operative frequencies for all the CRN users (PUs and SUs) includes *K* frequencies $F=\{f_{1},f_{2},\ldots ,f_{j},\ldots ,f_{K}\}$. In addition to this, the model includes a low bandwidth communication channel from the unlicensed band which is used for SU control signalling communication (CSC). The payload data packets between the communicating couple of radios *i*, formed of one SU transmitter (which we denote as ${\mathrm{SU}}_{i}$) and one SU receiver (${\mathrm{RSU}}_{i}$), are transmitted over the *K* licensed channels. Primary user traffic for each of the *K* channels is modeled as a two state birth-death process, with death rate $\alpha_{j}$ and birth rate $\beta_{j}$ associated to channel *j*. Modeled this way, the transitions of PU activity from state to state (i.e., ON to OFF) in a channel follow a Poisson process with exponentially distributed inter-arrival times. Posterior probabilities of channel use by the PUs can, thus, be estimated as:

(1) $$p_{j}^{{\mathrm{PU}}_{\mathrm{on}}}=\beta_{j}/\left(\alpha_{j}+\beta_{j}\right),\;p_{j}^{{\mathrm{PU}}_{\mathrm{off}}}=\alpha_{j}/\left(\alpha_{j}+\beta_{j}\right),$$

where $p_{j}^{{\mathrm{PU}}_{\mathrm{on}}}$ and $p_{j}^{{\mathrm{PU}}_{\mathrm{off}}}$ are the posterior probabilities that the channel *j* is occupied by the PU, i.e., PU is transmitting or using the channel (frequency) *j*, and that the channel *j* is vacant, i.e., that the PU is idle, respectively [35]. Packet error rate (PER) is considered per each channel; let ${\mathrm{PER}}_{j}$ be the PER in channel *j*. In addition, three actions can be performed per time-slot by a transmitting ${\mathrm{SU}}_{i}$:

- SENSE, whereas the ${\mathrm{SU}}_{i}$ senses the frequency to which it is currently tuned in order to determine the presence of PU activity. A default energy-detection sensing scheme is assumed [5], with $p_{D}$ indicating the probability of correct detection and $1-p_{D}$ the probability of sensing errors;
- TRANSMIT, whereas the ${\mathrm{SU}}_{i}$ tries to transmit one packet to the ${\mathrm{RSU}}_{i}$, while implementing the Carrier Sense Multiple Access (CSMA) as Media Access Control (MAC) protocol. Transmission is attempted until an acknowledgement packet is received or a maximum number of attempts (MAX_ATT) is reached, in which case the packet is discarded;
- SWITCH, in which case the ${\mathrm{SU}}_{i}$ of the pair switches to a different licensed frequency and notifies ${\mathrm{RSU}}_{i}$ of the switch via the CSC channel.

Time sequence of slots for ${\mathrm{SU}}_{i}$ can be described as $T_{i}=\left(\tau_{i}^{0},\tau_{i}^{1},\tau_{i}^{2},\ldots \right)$, where $\tau_{i}^{k}$ is the *k*-th time slot implemented by the ${\mathrm{SU}}_{i}$ and its values, based on the actions taken in it, can be $\tau_{i}^{k}\in \left\{\mathrm{SENSE},\mathrm{TRANSMIT},\mathrm{SWITCH}\right\}$. Constraints adhered to by the ${\mathrm{SU}}_{i}$, while making decisions on its schedule of actions $T_{i}$, are: (1) if $\tau_{i}^{k}=\mathrm{SENSE}$ and the channel is found to be busy, then $\tau_{i}^{k+1}\neq \mathrm{TRANSMIT}$ (agent cannot transmit on a channel occupied by PU), and (2) if $\tau_{i}^{k}=\mathrm{SWITCH}$, then $\tau_{i}^{k+1}=\mathrm{SENSE}$, meaning that the agent needs to establish the state of occupancy of a channel upon switching to it prior to trying any transmissions. The counters and outcomes of individual transmissions considered in the model include the total numbers of: transmissions, successful transmisssions, transmissions failed due to collision with PU and transmissions failed due to other channel errors. Having formulated the model thusly, the problem we are solving is related to a search for the optimal schedule of transmissions $T_{i}$ for each ${\mathrm{SU}}_{i},i=1,\cdots ,N$.

### 3.2. Reinforcement Learning and Markov Decision Processes

Before we formulate the above JSS model in an appropriate form for the application of RL algorithms, we first introduce a general problem setup used in RL. RL is based on a paradigm of learning by trial-and-error through interactions with the (unknown) environment. In general, each action of an agent influences the current state of the environment and brings a (possibly random) reward for that particular action and state transition. The aim of the agent is to discover a policy of behavior that maximizes the expected long-term sum of rewards gained for the actions taken [8].

Discrete Markov decision process (MDP) can be used to model the system underlying a typical RL problem setup. MDP can be represented by a tuple (S,A,R,T), in which:

- S represents a discrete set of available states; we denote the current state of an agent at a discrete time *k* as s(k),
- A represents a discrete set of available actions; we denote the set of actions available in state s(k) as A(s(k)),
- R:S×A→R is the reward function representing a numerical reward (or average reward in case of random rewards) received after applying an action at a certain state; let r(k) indicate the (possibly random) reward received by the agent while being in state s(k) and executing action a(k)∈A(s(k)),
- T:S×A→S is the state transition function, which indicates the next state s(k+1) after executing action a(k)∈A(s(k)) in state s(k); in case of nondeterministic environments, the *T* function is a probability distribution over the set of actions and states, i.e., T:S×A×S→[0,1].

We can further define a policy function π:S→A, which indicates an action a(k) to be performed at a state s(k). This function can also be modeled as a probability distribution over a set of actions and states π:S×A→[0,1] (randomized policy). Having defined the policy, the mentioned goal of RL of an agent can be expressed as the discovery of the optimal policy $\pi^{*}$ which would maximize a certain function of the rewards received for the actions taken over time. Future rewards of an agent can be discounted in order to model the agent impatience. The goal of RL of an agent can then be described as:

(2) $$\mathrm{find}\;\pi^{*}\;\mathrm{maximizing}\;E_{\pi }\left[\sum_{k=0}^{\infty }\gamma^{k}r\left(k\right)\right],$$

where $E_{\pi }$ denotes the mathematical expectation with respect to the Markov chain induced by policy π, while 0≤γ≤1 is the factor of discount for future rewards; if γ=0, the agent maximizes only the immediate rewards, while, for γ=1, the agent maximizes the sum of all the rewards received (which is feasible only if an absorbing state with zero reward exists). Two data functions can be used for computation of the optimal policy. The state-value function $v^{\pi }\left(s\right)$ represents the expected reward when following policy π starting from state *s* and (when the model of the environment is known) can be calculated using Bellman equation [8]:

(3) $$\begin{array}{c} v^{\pi }\left(s\right)=\sum_{a\in A(s)}\pi \left(s,a\right)\sum_{s^{\prime }\in S}T\left(s,a,s^{\prime }\right)\times \left(R\left(s,a\right)+\gamma v^{\pi }\left(s^{\prime }\right)\right). \end{array}$$

The action-value function $Q^{\pi }\left(s,a\right)$ represents the expected reward when an agent executes an action *a* from the state *s* and then continues with the policy π. It can be calculated as:

(4) $$\begin{array}{c} Q^{\pi }\left(s,a\right)=\sum_{s^{\prime }\in S}T\left(s,a,s^{\prime }\right)\times \left(R\left(s,a\right)+\gamma v^{\pi }\left(s^{\prime }\right)\right). \end{array}$$

For the optimal *Q* function (under optimal policy), the following holds:

(5) $$\begin{array}{c} Q^{*}\left(s,a\right)=\sum_{s^{\prime }\in S}T\left(s,a,s^{\prime }\right)\times \left(R\left(s,a\right)+\gamma \max_{a^{\prime }}Q^{*}\left(s^{\prime },a^{\prime }\right)\right). \end{array}$$

If the model of the environment (i.e., the state transition and reward functions) is unknown, the model-free RL techniques are used for evaluating a policy or for finding the optimal one. Equation (3) represents the basis of the so-called TD learning algorithm, whereas Equation (5) represents the basis of the so-called Q-learning algorithm [8]. Distributed versions of both of these algorithms will be proposed in the next section.

### 3.3. MDP Formulation of the JSS Model

We are now ready to formulate the above presented JSS model using the appropriate MDP formulations, suitable for applications of RL algorithms:

- Learning agents are the ${\mathrm{SU}}_{i}$ entities, i=1,…,N.
- We denote the current state of an agent ${\mathrm{SU}}_{i}$ at a discrete time *k* as $s_{i}\left(k\right)$, i=1,…,N.
- The set of states S is a set of couples $\left(f_{j},{\mathrm{AVAIL}}_{j}\right)$, where $f_{j}$ is a frequency from the set *F* (of *K* licensed frequencies or channels) and ${\mathrm{AVAIL}}_{j}=\left\{\mathrm{IDLE},\mathrm{BUSY},\mathrm{UNKNOWN}\right\}$ represents the sensed state of availability of channel *j*.
- The set of actions for an agent *i*, i.e., ${\mathrm{SU}}_{i}$, is $A_{i}=\left\{\mathrm{SENSE},\mathrm{TRANSMIT},{\mathrm{SWITCH}}_{f_{1}},\ldots ,{\mathrm{SWITCH}}_{f_{j}},\ldots ,{\mathrm{SWITCH}}_{f_{K}}\right\}$, i=1,…,N, j=1,…,K, where ${\mathrm{SWITCH}}_{f_{j}}$ is the action of switching to the frequency $f_{j}$, whereas the agent does not switch to the frequency it is currently tuned to.
- The reward function R:S×A→PMF[0,1], where PMF[0,1] is a probability mass function defined according to the actions taken and states encountered, i.e.,
  $$\begin{array}{cc} \; & R\left(\left(f_{j},*\right),\mathrm{SENSE}\right)=\zeta ,\mathrm{if}\;f_{j}\;\mathrm{is}\;\mathrm{found}\;\mathrm{IDLE},\\ \; & R\left(\left(f_{j},*\right),\mathrm{SENSE}\right)=0,\mathrm{if}\;f_{j}\;\mathrm{is}\;\mathrm{found}\;\mathrm{BUSY},\\ \; & R\left(\left(f_{j},\mathrm{IDLE}\right),\mathrm{TRANSMIT}\right)=1-\frac{\#\mathrm{retrans}}{\mathrm{MAX}\_\mathrm{ATT}},\\ \; & R(\left(f_{j},\mathrm{UNKNOWN}\right),\mathrm{SWITCH})=0, \end{array}$$
  where ζ is a scalar parameter associated with the model design (as explained in Section 5), and #retrans is the number of packet retransmissions performed, which is a random variable dependent on the PER.
- The state transition function T:S×A×S→[0,1] is defined as:
  (6) $$\begin{array}{cc} \; & T\left(\left(f_{j},*\right),\mathrm{SENSE},\left(f_{j},IDLE\right)\right)=\alpha_{j}/\left(\alpha_{j}+\beta_{j}\right),\\ \; & T\left(\left(f_{j},*\right),\mathrm{SENSE},\left(f_{j},BUSY\right)\right)=\beta_{j}/\left(\alpha_{j}+\beta_{j}\right),\\ \; & T(\left(f_{j},IDLE\right),\mathrm{TRANSMIT},\left(f_{j},IDLE\right))=1,\\ \; & T(\left(f_{j},*\right),\mathrm{SWITCH},\left(f_{k},UNKNOWN\right))=1. \end{array}$$
  The state transition function value is 0 for all the other argument values. Note that, as often is the case in practice, the channel state switching from IDLE to BUSY (or BUSY to IDLE) happens with a frequency far smaller than the learning rate of the SUs; in this case, it is possible to set, during these time intervals, the probabilities of sensing IDLE, or BUSY, to either 0 or 1. See the simulations section for more details.

**Figure 1:**
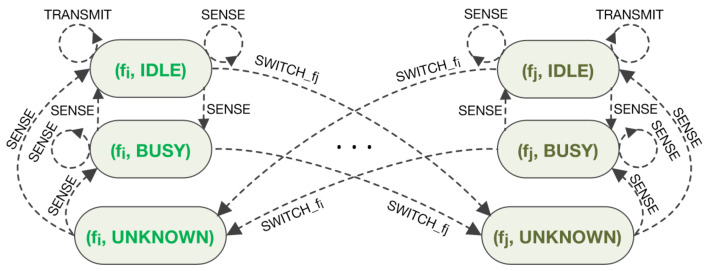
JSS problem formulation presented as MDP.

Figure 1 presents the MDP diagram for the described JSS problem illustrating only states and actions related to frequencies $f_{i}$ and $f_{j}$ out of the set of all *K* frequencies.

## 4. Consensus-Based Distributed Joint Spectrum Sensing and Selection

In this section, we propose novel, truly decentralized and distributed, networked MARL solutions to the problem of JSS in CRNs, which exploit possibility of cooperation among neighboring nodes, while preserving scalability and robustness properties. Specific adaptations and extensions of the MARL schemes presented in References [12,19,20] are proposed to tackle the JSS problem. The proposed decentralized scheme can be regarded through three main aspects: (1) as a tool for organizing coordination of actions of multiple nodes/agents, covering complementary parts of the state space, but contributing to a common goal, (2) as a parallelization tool, allowing faster convergence, useful particularly in the problems with large dimensions (e.g., large number of available frequency channels), and (3) as a denoising tool, exploiting a possibility to average agents’ different noise realizations, including the cases in which some agents may have large spectrum sensing probabilities of error, or are faced with higher PER on certain channels (e.g., due to the fading and shadowing effects); in such cases, their decisions will be, in average, corrected by typically larger number of nodes with better sensing conditions.

Specifically, we focus on a MARL setting where *N* autonomous SUs/nodes/agents are connected through a dedicated (typically low bandwidth) network (independent or dependent on the CSC) and are able to communicate information in real time only with the neighboring nodes (e.g., mobile ad hoc networks and sensor networks [1,28,36,37]). We formally model this dedicated communication network by a directed graph G=(N,E) where N={1,…,N} is the set of nodes, and E={(i,j)} the set of directed links (i,j). Denote as $N_{i}$ the set of neighboring nodes of the node *i* (i.e., the set of nodes which can send information to the node *i*, including node *i* itself). For large scale networks, it is typically expected that $|N_{i}|\ll N$.

Each ${\mathrm{SU}}_{i}$ is operating within the MDP described in the previous section. They are acting according to their local policy, applying their local actions (sense, transmit or switch to other channel), getting responses of the environment to their actions, and receiving local rewards corresponding to specific MDP transitions (reflecting presence of PU and quality of transmission over the used licensed spectrum, according to the above problem setup). In this problem description, it is assumed that the nodes do not interact with each other through the underlying MDP, i.e., it is assumed that MDP transitions induced by each node’s actions are independent. In practice, this can be achieved, e.g., by a specific distribution of channels (introducing restrictions on actions of particular nodes), ensuring that inter-SUs interference is sufficiently low.

We define time instances *k* as a union of all time instances $T_{i}$ in which SUs make sequential decisions and induce MDP transitions.

### 4.1. Distributed Consensus-Based Policy Evaluation

In this subsection, we treat the problem of distributed policy evaluation in the above described multi-agent setting. It is assumed that each ${\mathrm{SU}}_{i}$ has a different behavior policy $\pi_{i}$ so that each MDP (corresponding to each ${\mathrm{SU}}_{i}$) reduces to a plane Markov chain with a different state transition matrix $P^{\left(i\right)}$ which is obtained from function *T* by fixing the policy to $\pi_{i}$. We consider the problem of decentralized evaluation of a particular target policy π (inducing a Markov chain with the transition matrix *P*). The value function of policy π is given in Equation (3). Hence, each agent seeks to learn the vector $V^{\pi }\in R^{3K}$ (since the total number of states is 3K, see the assumed model above). Let the Markov chain under the target policy π be irreducible, for which there exists a limiting distribution $D\in R^{3K}$, and a unique value function $V^{\pi }$. For our concrete model, this implies that, under the target policy π, the agents should “visit” each channel infinitely often (see the next section for discussion on relaxation of this condition). We further introduce the local importance sampling ratios $\rho_{i}\left(s,s^{\prime }\right)=P_{ss^{\prime }}/P_{ss^{\prime }}^{\left(i\right)}$ for all $s,s^{\prime }\in S$ (with 0/0=0), where $P_{ss^{\prime }}$ and $P_{ss^{\prime }}^{\left(i\right)}$ are the probabilities of transiting from state *s* to $s^{\prime }$ under π and $\pi_{i}$, respectively. We denote each agent’s estimate of the value function vector by $V_{i}\in R^{3K}$. Introduce the global vector of all the agents’ estimates by $V_{G}={\left[V_{1}^{T}\cdots V_{N}^{T}\right]}^{T}$.

Based on the results from Reference [12], we define a global constrained minimization problem for the whole network, indicating how closely the estimates of the value function satisfy the Bellman equation:

(7) $$\begin{array}{cc} & J\left(V_{G}\right)=\sum_{i=1}^{N}q_{i}J_{i}\left(V_{i}\right)\\ & \mathrm{subject}\;\mathrm{to}\;\;V_{1}=\ldots =V_{N}, \end{array}$$

where $J_{i}\left(V_{i}\right)={∥V_{i}-\left(R+\gamma PV_{i}\right)∥}_{D_{i}}^{2}$ are the *local objective functions*, $q_{i}>0$, i=1,…,N, a priori defined weighting coefficients, and $D_{i}\in R^{3K}$ invariant probability distributions of each agent’s Markov chain (induced by the local behavior policies), *R* is the vector of the expected immediate rewards (for all states).

Based on the above setup, we propose a distributed and decentralized consensus-based algorithm for the estimation of $V^{\pi }$ aimed at minimization of (7), which is an adaptation to our specific problem of the general algorithm proposed in References [11,12]. The scheme is based on a construction of a distributed stochastic gradient scheme, resulting in the TD+consensus iterations:

(8) $$\begin{array}{cc} V_{i}{\left(k\right)}^{\prime (s_{i}\left(k\right))}= & V_{i}{\left(k\right)}^{\left(s_{i}\left(k\right)\right)}+\alpha q_{i}\rho_{i}\left(k\right)\delta_{i}\left(k\right) \end{array}$$

(9) $$\begin{array}{cc} V_{i}\left(k+1\right)= & \sum_{j\in N_{i}}c_{ij}\left(k\right)V_{j}{\left(k\right)}^{\prime }, \end{array}$$

where α is (typically small) step size, $\delta_{i}\left(k\right)=r_{i}\left(k\right)+\gamma V_{i}^{\left(s_{i}{\left(k\right)}^{\prime }\right)}-V_{i}^{\left(s_{i}\left(k\right)\right)}$ is the TD error, $V_{i}^{\left(s\right)}$ and $V_{i}^{\prime (s)}$ denote the value function estimates at state *s* before and after local update (8), respectively, $r_{i}\left(k\right)$ is the reward received by node *i* at step *k*, $s_{i}\left(k\right)$ is the state of ${\mathrm{SU}}_{i}$ at time *k*, $s_{i}{\left(k\right)}^{\prime }=s_{i}\left(k+1\right)$, and $\rho_{i}\left(k\right)$ is the importance sampling ratio at step *k* for agent *i*. The initial conditions for the recursions are arbitrary. The parameter $c_{ij}\left(k\right)$ represents the gain at step *k* which agent *i* uses to weight the estimates received from agent *j* (note again that this communication takes place through the dedicated, signaling network defined above, and not through the licensed spectrum being explored by the SUs). These parameters are random in general; they are equal to some predefined constants if the consensus communication at step *k* succeeded (with probability $p_{c}^{\left(i\right)}\left(k\right)$), and equal to zero if the communication failed (with probability $1-p_{c}^{\left(i\right)}\left(k\right)$). In addition, $c_{ij}\left(k\right)\equiv 0$ if $j\notin N_{i}$, i.e., if node *j* is not a neighbor of node *i*.

The algorithm consists of two steps: (1) local parameter updating (8) based on a locally observed MDP transition and a locally received reward; (2) consensus step (9) at which the local neighbors-based communication happens (along the dedicated network). The second step is aimed at achieving (in the decentralized way) the global parameter estimation based on distributed agreement between the agents, see Algorithm 1 for the implementation details.

In References [11,12], the convergence of the above algorithm has been proved under several general assumptions. For our specific problem setup, the convergence of the above algorithm is guaranteed under the following conditions on the agents behavior policies:

(A1) The transition matrices $P^{\left(i\right)}$ are irreducible and such that for all $s,s^{\prime }\in S$, $P_{ss^{\prime }}^{\left(i\right)}=0$⇒$P_{ss^{\prime }}=0$, i=1,…,N.

The condition of irreducibility essentially means that the underlying MDPs (under all the behavior policies) are such that all the agents should be able to explore all the states. The second part ensures that the importance sampling ratios $\rho_{i}\left(k\right)$ are well defined. Note that, in practice, according to our multi-agent setup, each agent is typically focused only on a part of the overall spectrum, by assigning high probabilities of visiting these parts of the state (channels), and low probability to the others. This complementarity can drastically improve the overall rate of convergence, as will be demonstrated in the simulations section.

The following conditions deal with the inter-node communication structure:

(A2) There exist a scalar $p_{0}>0$ and an integer $k_{0}$ such that

$\mathrm{Prob}\{{\mathrm{SU}}_{j}\;\mathrm{communicates}\;\mathrm{to}\;{\mathrm{SU}}_{i}\;\mathrm{on}\;\mathrm{interval}$$\left[k,k+k_{0}\right]\}\geq p_{0}$, for all *k*, and i=1,…,N, $j\in N_{i}$.

(A3) The digraph G is strongly connected.

(A4) Random sequences $c_{ij}\left(k\right)$, i,j=1,…,N (consensus communication gains) are independent of the Markov chain processes induced by the agents’ behavior policies.
**Algorithm** **1:** Distributed consensus-based policy evaluation.
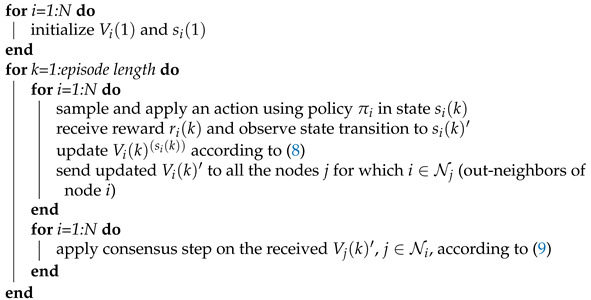


Assumption (A2) formally requires existence of a finite upper bound (uniformly in *k*) on the duration of any time interval (number of iterations) in which the neighboring SUs are not able to communicate with positive probability of communication success. Hence, it allows a very wide class of possible models of communication outages, such as the randomized gossip protocol with the simple Bernoulli outage model [38]. (A3) defines the minimal inter-agent network connectivity. The requirement is that there is a path between each pair of nodes in the digraph, which is needed to ensure the proper flow of information through the network (see, e.g., References [38,39]). Assumption (A4) is, in general, required for successful stochastic convergence, i.e., for proper averaging within the consensus-based schemes [12]. We consider all the conditions to be logical and typically satisfied in practice.

Careful selection of weighting factors $q_{i}$ and $c_{ij}\left(k\right)$ enables the user to emphasize contribution of certain nodes which have larger confidence in correct sensing of PU activities at certain channels compared to the remaining nodes. Furthermore, significant improvement of the overall rate of convergence of the algorithm can be achieved by a proper design strategy that would facilitate a form of overlapping decomposition of the global states (frequencies) leading to complementary nodes’ behavior policies. Another point to be considered are the time constants of information flow throughout the network. Implementation of multi-step consensus among the nodes within time intervals between successive measurements might be beneficial in cases when the possible inter-nodes communication rates are larger, allowing such a scheme [37]. Even if the agents’ behaviors are not selected in a complementary fashion, i.e., the visited states largely overlap among the agents, the “denoising” phenomenon represents another motivation for adopting the proposed consensus-based approach. In general, estimation algorithms based on consensus are characterized by nice “denoising” properties, i.e., by reduction of the asymptotic covariance of the estimates [40,41]. Recall that the variance reduction is one of the fundamental problems in TD-based algorithms, in general, e.g., References [8,42], and that in References [11,12], wherein the denoising effect was proved for consensus-based schemes similar to the above proposed.

### 4.2. Distributed Consensus-Based Q-Learning

The policy evaluation scheme described in details in the previous subsection naturally generalizes to the case of the action-value function (Q-function) learning, from which an optimal policy can be directly obtained. The popular Q-learning algorithm [8] is a single-agent algorithm, derived from (5) by applying TD-based reasoning, similar to the state-value function TD-based learning in (8). Since the objective is now to find the optimal policy, the main difference is that, in each step, an action is typically not selected based on a fixed policy (as in the policy evaluation problem), but by applying some exploration/exploitation strategy.

Hence, for the purpose of distributed searching of optimal policy in our CRN JSS model, we propose to use the same setup as in the previous subsection, while replacing local iterations in (8) with the local Q-learning iterations:

(10) $$\begin{array}{cc} Q_{i}^{\prime }{\left(k\right)}^{\left(s_{i}\left(k\right),a_{i}\left(k\right)\right)}= & Q_{i}{\left(k\right)}^{\left(s_{i}\left(k\right),a_{i}\left(k\right)\right)}+\alpha q_{i}\left(r_{i}\left(k\right)+\gamma \max_{a}Q_{i}{\left(k\right)}^{\left(s_{i}{\left(k\right)}^{\prime },a\right)}-Q_{i}{\left(k\right)}^{\left(s_{i}\left(k\right),a_{i}\left(k\right)\right)}\right), \end{array}$$

(11) $$\begin{array}{cc} Q_{i}\left(k+1\right)= & \sum_{j\in N_{i}}c_{ij}\left(k\right)Q_{j}^{\prime }\left(k\right), \end{array}$$

where $Q_{i}\left(k\right)$ and $Q_{i}^{\prime }\left(k\right)$ are the matrices of *i*-th node’s estimates (before and after the local update (10) is applied, respectively) of the Q values (5) for all the possible state-action pairs, $Q_{i}{\left(k\right)}^{\left(s,a\right)}$ is the *i*-th node’s estimate of the optimal action value (after the consensus update has been applied) for the particular state-action pair (s,a), and α and $q_{i}$ are the same as in the previous section. Hence, in each time step, an agent performs the local iteration (10), locally updating only the estimate of the Q-function for the current state $s_{i}\left(k\right)$ and applied action $a_{i}\left(k\right)$, receives the estimates of all the Q values from its neighbors, and performs the consensus iteration on Q estimates for all the state-action pairs. The initial conditions can be set arbitrary; however, it should be kept in mind that high values of the initial conditions will encourage exploration if a greedy policy is applied. It is also possible to reset initial conditions once the first reward is received for a particular state-action pair [8].

In the typical Q-learning setup, the action at step *k* is chosen using a form of ϵ-greedy strategy [8]. In our multi-agent case, we propose a modified ϵ-greedy strategy, where, for the overall performance, it is beneficial that each agent, when exploring (with probability ϵ) has a different set of channels of preference. This way the convergence speed can be drastically improved by exploiting the complementary state space coverage by different agents (similar to the case of complementary behavior policies in the above case of policy evaluation). This has been demonstrated in the simulations section.

The consensus step in the algorithm is the same as in (9), except that the agents must communicate, in each iteration, their Q-function estimates for all the pairs of possible states and actions (see Algorithm 2 for details). A similar general scheme has been proposed in Reference [19], with rigorous convergence analysis, but under a considerably limiting assumption that the actions selection strategy is a priori fixed and independent of the current agents’ Q-function estimates.

All the other appealing properties discussed in the previous sections still hold for the above proposed distributed Q-learning algorithm.

### 4.3. Convergence Rate and Complexity Analysis

As has been discussed in, e.g., Reference [43], the non-asymptotic rate of convergence (the rate at which the "mean-path" of the TD or Q-learning algorithm converges) is exponential. The asymptotic rate of convergence can be analyzed by deriving asymptotic stochastic differential equation which shows a direct dependence of the asymptotic covariance of the value function estimate on the network connectivity [12]. In the consensus literature, the network connectivity is typically characterized using the so-called algebraic connectivity, i.e., the second smallest eigenvalue of the underlying graph Laplacian [39]. Obviously, the higher the algebraic connectivity, the higher is the asymptotic convergence rate, and the denoising effect of consensus is stronger (see References [12,41] for more details).

The computational complexity of the policy evaluation algorithm (8) and (9) is O(K) since in, each step, the algorithm updates 3K scalar values (value function estimates for each state). For the distributed Q-learning algorithm (10) and (11), the complexity is $O(K^{2})$ since the algorithm keeps track of the value of each state-action pair, which is less than 3K×(K+2). It should be emphasized that both algorithms are completely scalable with respect to network size, i.e., the computational complexity does not depend on the number of agents implementing the algorithm (provided that the underlying network is sparse, i.e., the average number of neighbors does not grow with the network size).
**Algorithm** **2:** Distributed consensus-based Q-learning.
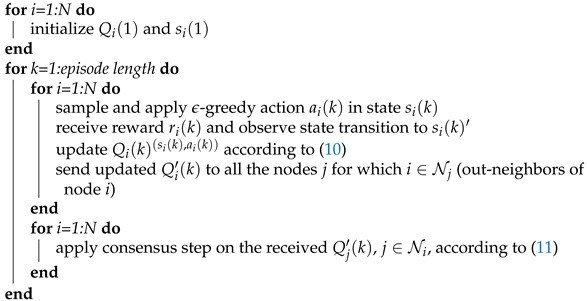


## 5. Simulations

In this section, we illustrate the discussed properties of the proposed consensus-based MARL JSS algorithms. We consider scenario with K=6 licensed channels, with their parameters given in Table 2; MAX_ATT=7. We assume N=6 learning agents (${\mathrm{SU}}_{i}$). We initially set ζ, the reward for sensing the idle state of the channel, to be 1. For simplicity, time duration of all state transitions is set to one time step of the simulation, which has been implemented in MATLAB software.

In the first experiment, the agents estimate the state-value function without knowledge of the model parameters, in off-policy setting, using the proposed Algorithm 1, with α=0.02 and γ=0.9. The adopted target policy with uniform probabilities for different types of actions, and with uniform probabilities for switching to different channels, is given in Table 3 (i,j=1,…,K,j≠i).

Behavior policies of different agents $\pi_{j}$, j=1,…,N, are similar to the target policy π, with one key difference: each agent has its channel of preference for which the switching probability is significantly higher than for the other channels. We have adopted the setting where agent *j* prefers channel $f_{j}$, with:

(12) $$\pi_{j}\left({\mathrm{SWITCH}}_{f_{j}}|\left(f_{i},\cdot \right)\right)=20\pi_{j}\left({\mathrm{SWITCH}}_{f_{k}}|\left(f_{i},\cdot \right)\right),$$

for i,j,k=1,…,K,j≠i,k≠i,j. The corresponding parameters are given in Table 4. In cases when the agents are already tuned to their channel of preference (j=i and $\pi_{j}\left({\mathrm{SWITCH}}_{f_{j}}|\left(f_{i},\cdot \right)\right)=0$), the probabilities for SENSE and TRANSMIT actions are scaled up so that $\pi_{j}\left({\mathrm{SWITCH}}_{f_{k}}|\left(f_{i},\cdot \right)\right)$ values would stay the same as stated in Table 4.

**Figure 2:**
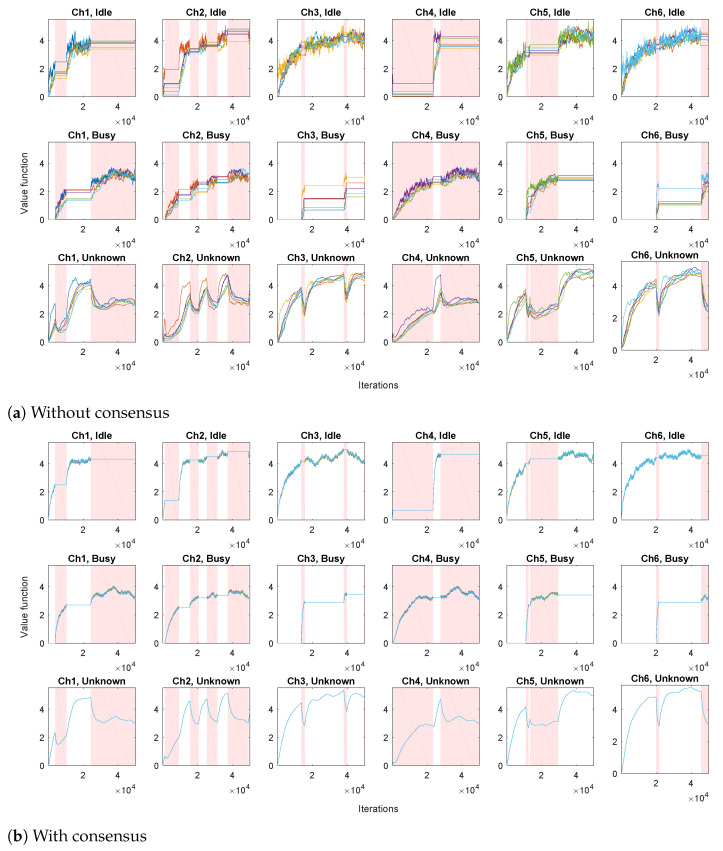
Value function estimations for all the states (different plots) and all the agents (different lines). Intervals during which the channels are busy are shown in shades of red.

We first evaluate a set of *N* independent TD algorithms, performing only the local TD step (8), without the consensus step (9). The obtained state-value function estimations are shown in Figure 2a. It can be seen that each channel has a corresponding agent that prefers switching to that channel, for whom the estimations climb more rapidly than estimations of the other agents (most notable in unknown states). In addition, the estimated values for the unknown states increase when the channels are idle and decrease when the channels are busy. The variation of the obtained estimations across different agents is considerable.

We then apply the proposed Algorithm 1, where in parallel with local TD algorithms (8), we have consensus iterations (9) (via communication scheme that exchanges local processing results between different agents). The ring communication topology is assumed, with each agent connected to 2 neighbors (e.g., agent 2 is connected to agents 1 and 3). Consensus weights $c_{ij}\left(k\right)$ are assumed to be time-invariant, and set to 1/3 for the connected agents, and to 0 otherwise. The resulting state-value function estimations are shown in Figure 2b. It can be seen that the variation of the estimates across different agents has been greatly reduced with respect to the case without consensus.

**Figure 3:**
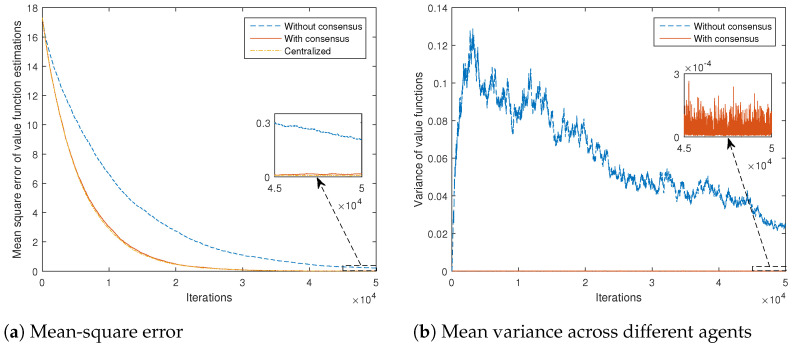
Error statistics of state-value function estimations for different algorithms, versus number of iterations.

In order to obtain a better quantitative comparison, mean-square error of value function estimations (with respect to the true values obtained by solving the corresponding Bellman expectation Equation (3) for the assumed model), averaged over all the states and agents, for the cases with and without consensus, is shown in Figure 3a. It is clear that the introduction of the consensus scheme significantly speeds up the convergence of the overall algorithm. The centralized scheme performance is also given, where all-to-all communications are assumed. It can be seen that the consensus scheme achieves results very close to those of the centralized scheme.

Mean variance of value function estimations across different agents, averaged over all the states, for the cases with and without consensus, is given in Figure 3b. It shows that the introduction of the consensus-based scheme reduces the variance of the estimates by approximately two orders of magnitude.

**Figure 4:**
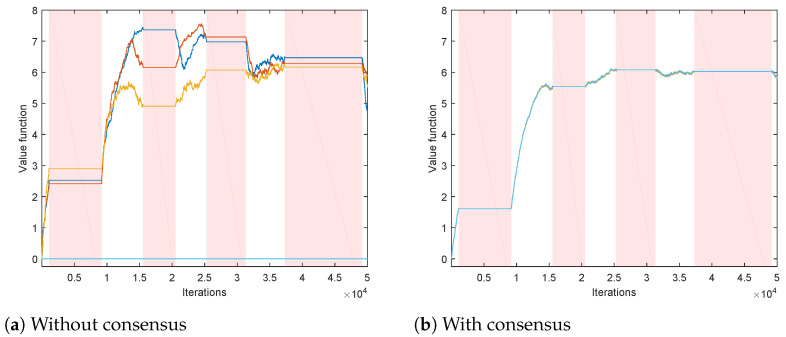
Value function estimations of all agents (different lines) for a state that can be visited by only three agents. Intervals when the corresponding channel is busy are shown in shades of red.

Consensus schemes are known to be able to provide viable estimations even to the agents not receiving observations regarding variables of interest. In order to illustrate this property, we use a setting where local behavior policies are such that each channel has 3 agents that can switch to it; other 3 agents never visit it. In this scenario, there are 3 “live” estimations for each local state-value function (example for one state is shown in Figure 4a); the other 3 are stuck at the initial values (we assumed zero initial conditions). Figure 4b affirms that the consensus scheme provides all the agents with viable estimations of the state-value functions in this scenario. It is to be noticed that, in this setting, part of the assumption (A1) related to the importance sampling ratios (the so-called assumption of coverage [8]) is clearly not satisfied. However, due to the introduced consensus scheme, the needed coverage is now achieved at the network level, enabling successful practical implementation of the Algorithm 1.

**Figure 5:**
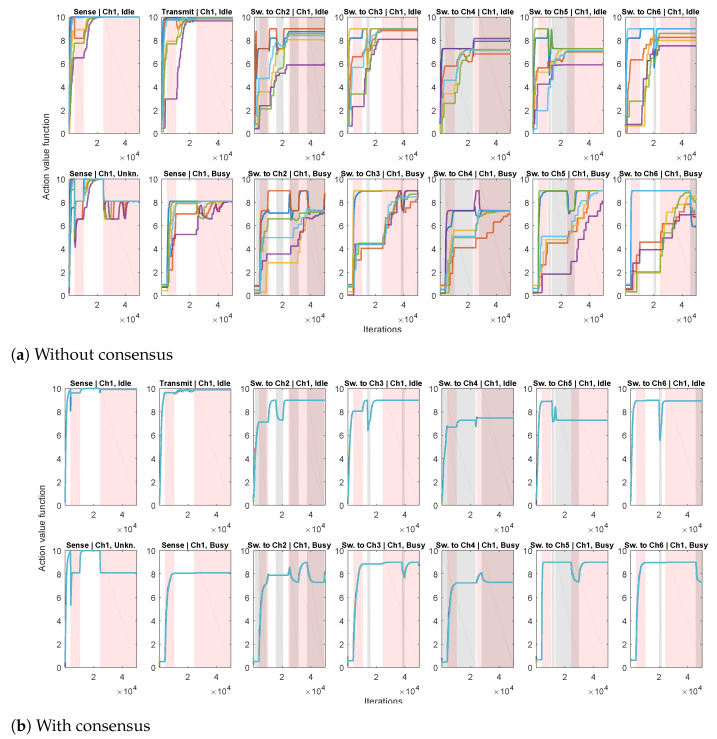
Action-value function estimations for all the possible actions from three states corresponding to a single channel (different plots with corresponding ActionjState pairs given in the titles) and all agents (differently colored lines). Intervals when the considered single channel is busy are shown in shades of red. Intervals when the channels corresponding to destinations of switching actions are busy are shown in shades of gray.

In the second experiment, we apply the proposed Algorithm 2, for estimating the action-value function (based on Q-learning (10) and consensus algorithm (11)), with α=0.2, γ=0.9 and $p_{D}=0.9$. Each agent implements its own ϵ-greedy behavior policy $\pi_{i}$, i=1,…,N, with ϵ=0.5. The setting is similar to the first experiment: each agent has its channel of preference when choosing exploratory actions, with 20 times greater probability of switching to its channel of preference than to the other channels. We first consider the case without consensus (only agents’ local processing (10), without communication with the other agents), illustrated in Figure 5a. We show the obtained Q-value estimates for actions corresponding to a single channel so that the figure doesn’t become too cluttered. It can be seen that, due to different exploratory policies, the agents’ action-value functions differ significantly. The case when the proposed consensus communication scheme is applied in shown in Figure 5b. Due to the beneficial properties of consensus, a high level of agreement between the agents has been achieved, so that the partially transparent lines of different colors corresponding to different agents’ estimates of the action-value mostly overlap (in each plot, there are N=6 overlapping lines). In addition, intervals corresponding to the availability of destination channels in case of switching actions are also depicted. It can be seen that the obtained action-value estimations follow channel availability conditions: they decrease when the destination channel (Dest.) is busy and increase when it is idle (in parts of Sw.toDest.|Ch1,Idle plots when Ch1 is idle and in parts of Sw.toDest.|Ch1,Busy plots when Ch1 is busy).

**Figure 6:**
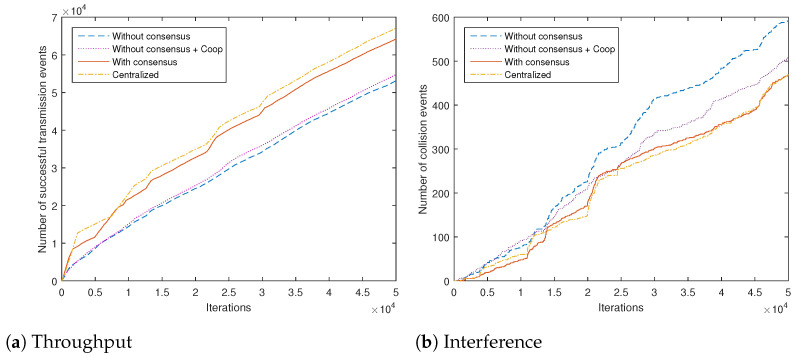
Total number of successful and failed transmission events in the network for different algorithms, versus number of iterations.

Towards obtaining some practically important measure of the algorithm performance with respect to the given task of the adopted JSS model, we count the number of successful and the number of failed transmissions (due to the interference with PUs) for different algorithms. Total network counts are shown in Figure 6a,b, respectively. We compare our proposed scheme with a baseline being a recently proposed representative decentralized cooperative scheme based on an aggregation of local rewards without consensus (algorithm from Reference [22] adapted to our system model, similar to the information dissemination scheme from Reference [2], labeled as "Without consensus + Coop"). It can be seen that the introduction of the proposed consensus scheme increases the throughput approximately by half in the first 10,000 iterations of the algorithm. The centralized scheme, as expected, does yield slightly better results. The selected baseline cooperative scheme performs worse than our consensus-based algorithm, due to its inherently higher variance in the Q-value estimates. The obtained numbers of successful and unsuccessful transmissions for the consensus-based scheme related to the individual channels are given in Table 5. It can be clearly seen that channel 6 (low PU activity and low PER) is the channel of preference for the transmitting actions.

It is to be emphasized that the illustrated beneficial properties of the applied consensus communication scheme, i.e., better coordination and lower disagreement between multiple agents, together with the increased convergence speed of the resulting algorithms, go beyond the adopted parametrization and the adopted system model. We have chosen this model, following Reference [2], in order to obtain a clear demonstration of the properties of the proposed scheme and the underlying action-value function estimates. There are many alternatives that can be used within the proposed consensus-based framework. Firstly, one may decrease ζ, the sensing reward in cases when the channel is found to be idle, which would, in a relative manner, raise the rewards for transmitting actions and, consequently, yield larger numbers in Table 5. Additionally, sensing errors may be taken into account more effectively by, e.g., adding penalty terms on rewards, in cases of transmitting actions on busy channels. In addition, the assumed PU activity model can be improved, resulting in better local processing results. We have performed experiments with different models obtained from historical statistics of PU activity [44]; the obtained comparative results are essentially the same as those presented in Figure 6a,b. It is also possible to use more advanced spectrum sensing approaches than the adopted energy detection scheme, such as machine learning-based [45]. However, even with the algorithms with improved local sensing, the obtained margin in difference of performances with and without consensus in Figure 6a would remain the same, since it is primarily influenced by the high variance of the estimates obtained by the algorithms without consensus.

## 6. Conclusions

In this paper, a novel consensus-based distributed algorithm has been proposed, within the multi-agent reinforcement learning paradigm, aimed at solving the JSS problem in CRNs. Both state-value function and action-value function learning algorithms have been covered, as potentially crucial steps towards obtaining the best possible JSS solution. It has been shown that the proposed algorithms enable successful achievement of the JSS goal by utilizing an efficient, typically sparse and completely decentralized coordination between the agents. The algorithms provide a capability to each agent of finding a globally optimal JSS policy even if the individual agents have limited, but complementary, channels coverage. The algorithms also provide faster convergence rate and lower noise of the estimated values of interest, compared to the corresponding basic single-agent schemes.

Extensions and future works include application of similar distributed actor-critic-based schemes in the CRN problems, including the scenarios with strong inter-SU interference. In addition, TD(λ) schemes offer interesting possibilities for the introduction of adaptive consensus schemes. Introduction of function approximations (for both state-value functions and action-value functions) in order to tackle very large state or state-action spaces is also in the future plans.

## Figures and Tables

**Table 1:** Overview of related work.

Authors	Problem Treated	Local Processing	Cooperative Scheme
C. Wu et al. [23]	Channel and power level selection	Q-learning	Centralized
T. Jiang et al. [24]	Efficient exploration in spectrum sharing	Linear value function approximation	None
Y. Tian et al. [13]	Channel and power level selection	MAB with policy index (energy efficiency)	None
N. Modi et al. [14]	Channel selection	MAB with learning policy (quality metric reflecting interference)	None
B.F. Lo, I.F. Akyildiz [1]	Cooperation overhead	Q-learning	Cooperative sensing
I. Mustapha et al. [25]	Cooperative channel sensing	Q-learning	Cluster heads aggregation
W. Ning [26]	Cooperative spectrum sensing	Q-learning	Partner selection algorithm
A. Kaur, K. Kumar [27]	Imperfect Channel State Information-based spectrum management	Q-learning	Assisted with cloud computing
S-J. Jang et al. [28]	Dynamic band and channel selection	Q-learning	Centralized
O. Naparstek, K. Cohen [29]	Distributed dynamic spectrum access	Deep Q-learning with LSTM	Centralized
S. Wang et al. [30]	Dynamic multichannel access	Deep Q-learning with Experience Replay	None
V. Raj et al. [31]	Channel selection and availability prediction	MAB setting and Bayesian learning	None
Y. Lin et al. [32]	Dynamic spectrum access, power allocation	Hybrid Spectrum Access Algorithm	None
A. Kaur, K. Kumar [22]	Dynamic spectrum management	Comparison-based Cooperative Q-Learning (CCopQL) and SARSA	Decentralized resource allocation
P. Yang et al. [33]	Dynamic spectrum access	Deep Q-learning	Balanced via replicator dynamic using evolutionary game theory

**Table 2:** Channel parameters.

Channel	1	2	3	4	5	6
α	10	5	2	10	5	2
β	2	5	10	2	5	10
PER	0.5	0.5	0.5	0.1	0.1	0.1

**Table 3:** Target policy parameters.

State/Action	SENSE	TRANSMIT	${\mathrm{SWITCH}}_{f_{j}}$
$\left(f_{i},\mathrm{IDLE}\right)$	1/3	1/3	1/15
$\left(f_{i},\mathrm{BUSY}\right)$	1/2	0	1/10

**Table 4:** Behavior policies parameters.

State/Action	SENSE	TRANSMIT	${\mathrm{SWITCH}}_{f_{j}}$	${\mathrm{SWITCH}}_{f_{k}}$
$\left(f_{i},\mathrm{IDLE}\right)$	1/3	1/3	5/18	1/72
$\left(f_{i},\mathrm{BUSY}\right)$	1/2	0	5/12	1/48

**Table 5:** Different channels’ contribution to the total number of successful and failed transmissions in the network.

Channel	1	2	3	4	5	6
successful transmissions	1440	2263	2885	115	11,739	45,678
failed transmissions	75	136	8	74	94	85

## Abbreviations

The following abbreviations are used in this manuscript:

CRN	Cognitive Radio Network
QoS	Quality of Service
CM	Cognition Module
DSA	Dynamic Spectrum Access
SU	Secondary User
PU	Primary User
CR	Cognitive Radio
RL	Reinforcement Learning
MDP	Markov Decision Process
MARL	Multi-agent Reinforcement Learning
DQN	Deep Q-Network
TD	Temporal Difference
JSS	Joint Spectrum Sensing and (channel) Selection
DCS	Dynamic Channel Selection
CSC	Control Signalling Communication
PER	Packet Error Rate
CSMA	Carrier Sense Multiple Access
MAC	Media Access Control
